# Targeting the parasite’s lifeline: knockout of SL transporters confers durable *Striga* resistance in sorghum

**DOI:** 10.1007/s44307-025-00062-y

**Published:** 2025-04-02

**Authors:** Jiayang Shi, Feifei Yu

**Affiliations:** 1 Institute of Genetics and Developmental Biology, The Innovative Academy of Seed Design, Chinese Academy of Sciences, Beijing, 100101 China; 2 College of Grassland Science and Technology, China Agricultural University, Beijing, 100193 China

## Introduction

In the arid farmlands of sub-Saharan Africa and Southeast Asia, each planting season witnesses a silent catastrophe. *Striga*—a parasitic plant known as “witchweed” due to its capability to drain life from cereal crops, affects over 40 million hectares of farmland, leading to annual losses exceeding $1 billion and trapping smallholder farmers in poverty (Gobena et al. 2017; Mutuku and Shirasu 2019). Traditional control methods like crop rotation or herbicides are ineffective because of the parasite’s long-lived seed bank and farmers’ limited resources. The core of this crisis is a molecular betrayal: strigolactones (SLs), signaling molecules used by crops to attract beneficial fungi, are hijacked by *Striga* as germination cues. For decades, researchers attempted to disrupt SL production, yet this approach damaged crop growth and ecological balance. Currently, Shi et al. redefine the strategy by focusing on SL transport rather than synthesis, achieving a breakthrough in parasite resistance without compromising crop health (Shi et al. 2025).

## The dual role of strigolactones

**Fig. 1:**
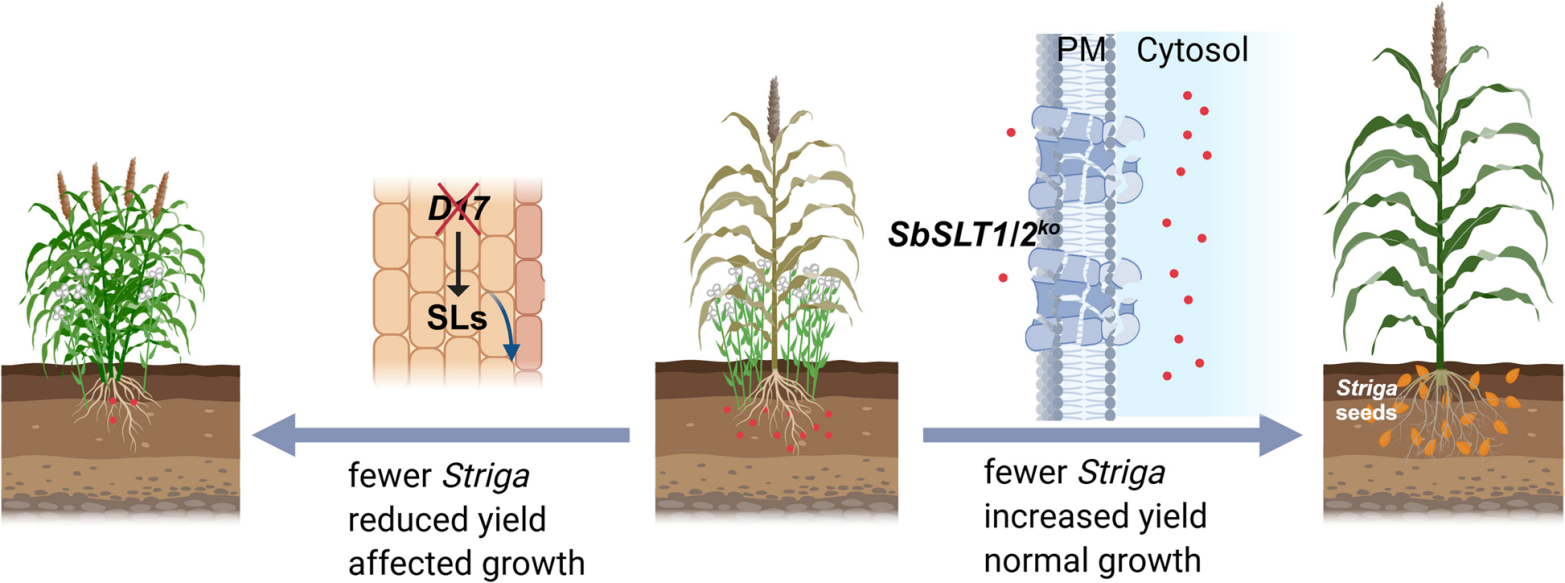
Crops resistance *Striga* through regulating SL biosynthesis or secretion. Left panel: Plants with knockout of *DWAR17*, which encodes *CAROTENOID CLEAVAGE DIOXYGENASE 7* (*CCD7*). CCD7 cleaves 9-cis-bcarotene into 9-cis-b-apo-10’-carotenal, a precursor of SL. Right panel: Plants with knockout of *SbSLT1/SbSLT2,* which encodes SL transporter in sorghum. The red points indicate SLs (modified from Shi et al., Cell, 2025)

SLs have a dual nature. Under phosphate-deficient conditions, plants release these molecules to recruit fungi that enhance nutrient uptake. However, these same signals can awaken dormant *Striga* seeds, which then invade roots using specialized structures called haustoria, stealing water and nutrients and releasing toxins. Farmers are confronted with a difficult choice: reducing SL production (e.g., via *SbD17* mutations) may decrease *Striga* infection but can also impede plant growth and reduce yields (Chen et al. 2018; Gomez-Roldan et al. 2008). Shi et al. addressed this conflict by focusing on SL transport rather than biosynthesis, thereby preserving crop vitality while inhibiting parasite germination (Fig. 1).

## Unlocking the secretion mechanism

The study commenced with a critical question: *How do SLs exit plant roots?* Although phosphate deficiency was known to boost SL production (Lopez-Raez et al. 2008; Yoneyama et al. 2013), Shi et al. hypothesized that specific transporters govern their release into the soil. Utilizing RNA sequencing, the team analyzed sorghum roots under phosphate-deficient conditions and GR24^5DS^ (a synthetic SL analog) treatment. Two ABCG-family transporters, SbSLT1 and SbSLT2, were identified as particularly active in root epidermal cells. Subsequent experiments in yeast, *Xenopus* oocytes, and *Arabidopsis* confirmed their function in SL export.

## Structural insights guide precision engineering

AlphaFold2 predictions unveiled the 3D structure of SbSLT1 and SbSLT2, revealing a shared hydrophobic channel for SL movement. A key phenylalanine residue (F693 in SbSLT1; F642 in SbSLT2) was identified as crucial for stabilizing SL molecules during transport. Replacing this residue with alanine disrupted SL export in yeast, confirming its essential function. Phylogenetic analysis demonstrated the conservation of this phenylalanine across diverse plant species, from petunia to tomato, indicating a universal transport mechanism (Ban et al. 2025; Kretzschmar et al. 2012). Importantly, analogous residues were found in maize, rice, and millet homologs, suggesting broad applicability in cereal crops.

## From Lab to field: CRISPR-edited sorghum triumphs

CRISPR-Cas9 knockout of *SbSLT1/2* decreased SL secretion by over 90% in hydroponic assays. Field trials in *Striga*-infested plots revealed striking outcomes: double mutants hosted 94% fewer parasites than wild-type sorghum across two growing seasons. Importantly, grain yields and biomass stayed unchanged in parasite-free fields, proving that inhibiting SL transport—not production—safeguards crop performance.

## Conclusion

Shi et al. present a landmark solution to *Striga* parasitism. By editing SL transporters instead of biosynthetic genes, they achieve resistance without compromising yields. The conserved phenylalanine residue offers a blueprint for engineering resistance in maize, rice, and other crops. For smallholder farmers, this innovation could break the cycle of poverty caused by *Striga*. Future research should focus on validating these results against the more aggressive *S. hermonthica* and assessing long-term ecological impacts. With further refinement, this strategy promises to transform parasitic weed management globally.
